# The neurobiology of schizotypy: Fronto-striatal prediction error signal correlates with delusion-like beliefs in healthy people

**DOI:** 10.1016/j.neuropsychologia.2012.09.045

**Published:** 2012-12

**Authors:** P.R. Corlett, P.C. Fletcher

**Affiliations:** a Connecticut Mental Health Center, Yale University, Department of Psychiatry, Ribicoff Research Facility. 34 Park Street, New Haven, CT 06519, USA; b University of Cambridge, School of Clinical Medicine, Addenbrooke's Hospital, Hills Road, Cambridge CB2 0SP, UK

**Keywords:** Schizotypy, Prediction error, fMRI, Delusions, Psychosis

## Abstract

Healthy people sometimes report experiences and beliefs that are strikingly similar to the symptoms of psychosis in their bizarreness and the apparent lack of evidence supporting them. An important question is whether this represents merely a superficial resemblance or whether there is a genuine and deep similarity indicating, as some have suggested, a continuum between odd but healthy beliefs and the symptoms of psychotic illness. We sought to shed light on this question by determining whether the neural marker for prediction error - previously shown to be altered in early psychosis – is comparably altered in healthy individuals reporting schizotypal experiences and beliefs. We showed that non-clinical schizotypal experiences were significantly correlated with aberrant frontal and striatal prediction error signal. This correlation related to the distress associated with the beliefs. Given our previous observations that patients with first episode psychosis show altered neural responses to prediction error and that this alteration, in turn, relates to the severity of their delusional ideation, our results provide novel evidence in support of the view that schizotypy relates to psychosis at more than just a superficial descriptive level. However, the picture is a complex one in which the experiences, though associated with altered striatal responding, may provoke distress but may nonetheless be explained away, while an additional alteration in frontal cortical responding may allow the beliefs to become more delusion-like: intrusive and distressing.

## Highlights

► People without psychosis nevertheless hold unusual, psychotic-like beliefs. ► Magical beliefs are associated with inappropriate striatal prediction errors. ► Distressing beliefs are associated with aberrant prefrontal prediction error. ► These findings partially support a continuum from healthy odd beliefs to delusions.

## 1 Introduction

Psychologically healthy people have an array of experiences, ideas and beliefs that may, on occasion, seem to overlap with those that characterize both emerging and established psychosis (David, 2010; Kretschmer, 1925). Research that has focused on identifying and quantifying these beliefs and relating them to psychiatric illnesses has inspired the growing idea that psychotic experience exists as a part of a continuum (Linscott & van Os, 2010). Clearly, clinical psychosis entails experiences and beliefs that are sufficiently intrusive and distressing to have a marked effect on individuals' quality of life and functioning. However, it can be extremely difficult to develop an operational way of determining what is or is not a part of normal experience, and it is correspondingly difficult to distinguish clearly between a belief that is truly delusional and one that is merely unusual, arcane or irrational (Peters, Day, McKenna, & Orbach, 1999b). It is a challenge to develop our understanding of these healthy but strange experiences and beliefs and their implications for our understanding of psychotic mental illness. Do high scores on schizotypy scales, which document these experiences, reflect an increased vulnerability to psychotic illness? Does the existence of the symptom-like experiences in the healthy population prove that psychosis lies on a continuum with normal mental function? Establishing the phenomenological similarity between psychosis and schizotypy will only provide partial answers to these questions. To characterize the relationship more fully, we suggest that it is important to determine whether there is overlap at the neurobiological level.

Our initial prediction, based on our own studies using purely behavioral measures (Corlett, Simons et al., 2009; Moore, Dickinson, & Fletcher, 2011b; Teufel, Kingdon, Ingram, Wolpert, & Fletcher, 2010) was that our fMRI observations would favor a continuum model. However, key to this study was the acknowledgment that it is possible to have a behavioral similarity while, nevertheless, the underlying neural basis of altered beliefs in schizotypy and in psychosis might be quite different. In this respect, we see the fMRI measure as a valuable, even an essential tool, in addressing fully questions such as this.

To that end, we sought to relate schizotypy to neural responses in an associative learning task. While undergoing functional magnetic resonance imaging (fMRI), healthy subjects completed a Kamin (1969) blocking task designed to reveal variations in patterns of prediction error (PE) signal across subjects. In blocking, prior learning leads to an attenuation of new learning such that there is a subsequently reduced expectation that a blocked stimulus has predictive power. Imagine that I have repeatedly learned that eating chicken causes an allergic reaction. If I eat a meal containing chicken and spinach, a subsequent allergic reaction is wholly predicted (by the presence of chicken in the meal) and I should develop no expectation that spinach has allergenic potential (in other words, learning about spinach has been “blocked”). If I now eat spinach alone and suffer an allergic reaction, this is relatively surprising: a prediction error results. This was the manipulation made in the current experiment. We made behavioral measures of expectancies as well as fMRI measures of neural responses during both blocking (low PE) and subsequent expectancy violation (high PE) trials in order to assay individual variability in blocking. Blocking was chosen because it enables a flexible characterization of PE signal (across low and high PE trials) and, moreover, it has already been explored behaviorally in healthy subjects whose responses are predictive of the severity of their attenuated psychosis-like experiences: for example, positive schizotypy scores on the OLIFE scale predict weaker blocking (Moore, Dickinson, & Fletcher, 2011a; Moran, Al-Uzri, Watson, & Reveley, 2003) – consistent with these attenuated positive symptoms forming under the influence of an aberrant learning process, like clinical delusions (Corlett, Murray et al., 2007).

Participants' schizotypal and related personality traits were quantified using the Chapman Scales (Eckblad & Chapman, 1983) and the Peters Delusion Inventory (PDI, Peters, Joseph, & Garety, 1999, see below). They then completed the blocking task during fMRI scanning. This entailed learning causal relationships between foods and allergic responses.

In prior work, we found that inappropriate dorsolateral prefrontal PE signal during causal learning in patients with psychosis was predictive of the severity of delusions (Corlett, Murray et al., 2007). Evidence that aberrant right frontal PE signal relates to schizotypy would therefore favor a continuum model of psychosis ranging from high schizotypy in health to delusional belief in psychotic illness (Johns & van Os, 2001). However, a lack of comparable relationship between inappropriate prediction error responding and schizotypal features would, we argue, call into question any simple idea that schizotypy can be seen as an attenuated form of psychosis. Rather, such a negative finding would be more consistent with a conception of high schizotypy as a phenocopy of clinical symptoms: phenomenologically similar but neurobiologically separable and to some degree distinct from psychosis (Meehl, 1989).

## 2 Methods

### 2.1 Subjects

Eighteen (eight female) right-handed, healthy volunteers were recruited through local advertisement for a combined functional imaging and psychopharmacology study in which they completed this causal learning task and other cognitive tasks in the fMRI scanner as well as a placebo controlled behavioral study on the effects of ketamine separated from the scanning session by at least a month (data reported elsewhere). No subjects reported a history of psychiatric illness, drug abuse or contra-indications for MRI. We excluded subjects with any history of alcoholism as well as current smokers (Domino, Mirzoyan, & Tsukada, 2004; Krystal et al., 2003; Petrakis et al., 2004). The study was approved by the Cambridge Local Research and Ethics Committee and was carried out in accordance with The Code of Ethics of the World Medical Association (Declaration of Helsinki). One subject was excluded upon discovery of a past history of psychiatric illness.

### 2.2 Schizotypy scales

There are numerous self-report rating scales that can be used to capture the various dimensions of schizotypal personality (Claridge & Beech, 1995). We chose the Chapman scales (Chapman, Chapman, & Raulin, 1976, 1978; Eckblad & Chapman, 1983) and the Peters Delusion Inventory (Peters et al., 1999) because they formed the basis of prior behavioral work relating aberrant PE and salience to schizotypy (Corlett, Simons et al., 2009).

### 2.3 Chapman scales

We administered the four scales developed by Chapman and colleagues to assess schizotypy: (1) The physical anhedonia scale (Chapman et al., 1976) consisting of 61 true –false items that measure a deficit in the ability to experience pleasure; (2) the social anhedonia scale (Chapman et al., 1976), 40-items, which tap social withdrawal and indifference to other people; (3) the perceptual aberrations scale (Chapman et al., 1978), 28-items that assess experience of the internal and external world that share surface similarities with many of the perceptual/attentional disruptions documented in the earliest phases of psychosis (Chapman, 1966) and (4) the magical ideation scale (Eckblad & Chapman, 1983), 30 items that assess endorsement of causal mechanisms that are invalid or metaphysical (e.g. telekinesis). Subjects completed all questions from these scales in a randomized order so as not to reveal or emphasize particular themes (Laurent et al., 2000). Given our thematic focus on delusions as aberrant causal inference (Corlett, Honey, & Fletcher, 2007), we were particularly interested in the magical ideation scale scores. However, for completeness, the scores on the other scales were included in our multiple regression analysis with PE neural responses (see below).

### 2.4 Peters Delusion Inventory (PDI)

Subjects completed the 21-item PDI with pen and paper (Peters, Joseph, Day, & Garety, 2004a). This scale was constructed to gather more information about the common and seemingly benign psychosis-like beliefs in the general population (van Os, Hanssen, Bijl, & Ravelli, 2000). Face validity with clinical delusions was assured by using the Present State Examination (PSE) delusional themes (Wing, Cooper, & Sartorius, 1974) as a template for constructing items. Items were adapted for healthy, non-psychotic individuals by prefacing items with a relative, “as if” extension (e.g. “*Does it ever feel as if…?*”). Furthermore, the PDI attempts to capture the multidimensionality of delusions; Peters claims: “*It is not what you believe but how you believe it*” (Peters et al., 2004a); as such, for every belief endorsed, subjects are required to fill out 5-point Likert scales that assess the degree of distress, pre-occupation and conviction associated with the belief. The degree of distress associated with a particular belief, rather than the total number of beliefs endorsed, distinguishes healthy non-clinical odd beliefs from clinical delusions (Peters, Day, McKenna, & Orbach, 1999a; Sisti et al., 2012)

The validity of the PDI was ascertained from its construction (it is based on the PSE); furthermore, PDI scores correlate with other measures of delusions (Peters, Joseph, Day, & Garety, 2004b) including the BPRS subscales pertaining to delusions (Kao, Wang, Lu, Cheng, & Liu, 2012), adding construct validity. In prior work, we used PSE and BPRS delusions scores to relate prediction error brain signal to drug induced and endogenous delusions (Corlett et al., 2006; Corlett, Murray et al., 2007). Furthermore, the relative “*as if*” statements are very similar to the phenomenological descriptions of first episode psychosis patients in the formative delusional-mood stage of their psychopathology (Gross & Huber, 1972) and healthy subjects administered a psychotomimetic dose of ketamine (Corlett, D'Souza, & Krystal, 2010). Hence, the PDI is particularly relevant for our current purposes.

Given the discriminant power of PDI distress scores, with regards to odd versus clinically relevant beliefs, distress scores formed the focus of our PDI analyses. However, total number of beliefs endorsed and participants' scores on the other dimensions were included in our multiple regression model relating PDI with PE brain responses (see below).

### 2.5 Functional neuroimaging of PE signal

**Figure 1:**
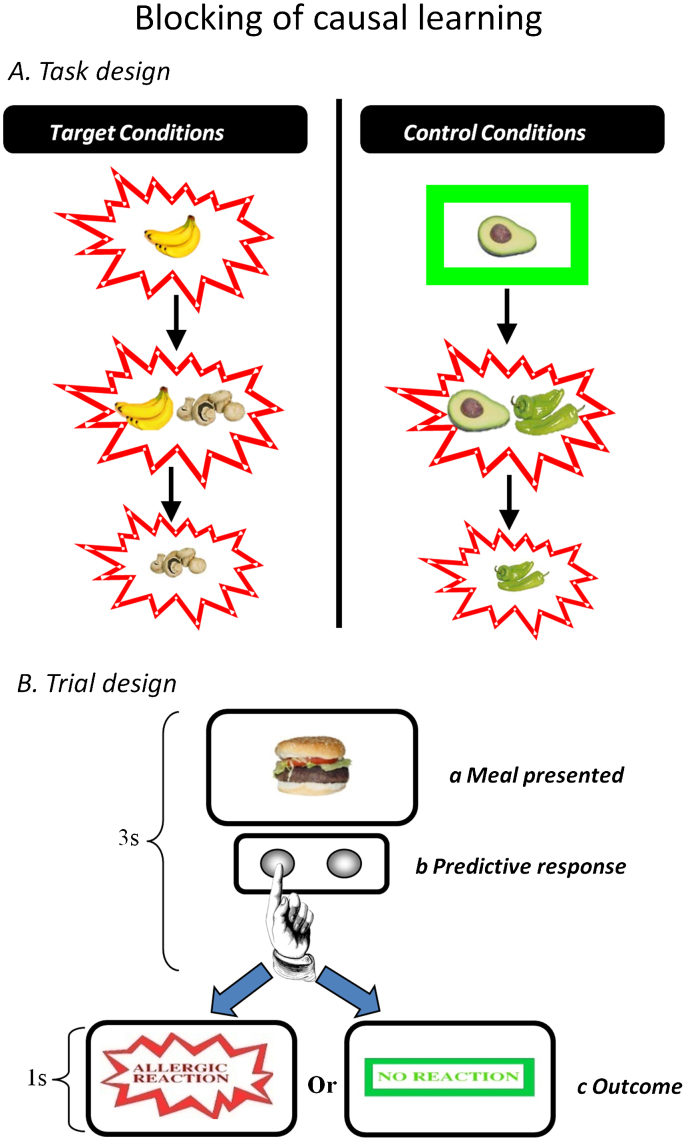
Study design. (a) Task Design. Target and control conditions for the food-allergy causal learning tasks. Subjects see that *bananas* cause an allergy in their patient. Subsequently they see that *bananas* and *mushrooms* cause the allergy. Their prior learning about bananas should block new learning about the mushrooms. In the final phase of training, subjects see the mushrooms causing the allergy; this violates any blocking that took place in the previous stage. Blocking trials are compared to control events that are matched for the presence of allergy as well as novelty and familiarity (*Avocado and Chillies*). Likewise, at stage 3, there were trials matched for novelty and familiarity that act as comparators for the blocking violation events. (b) Trial design. On each trial, subjects saw a meal that their patient had eaten for 3 s. During this time, they made a prediction response – pushing one button to predict an allergy and another to predict no allergy. They also held the button down for longer the more confident that they were making the right choice. Next they were shown the effect of that meal on their patient. If he suffered an allergy, they would see the words Allergic Reaction in red letters with a jagged border for 1 s. If there was no allergy, subjects saw the words no allergy in green letters with a green rectangle around it for 1 s.

We used an established causal learning approach (Corlett et al., 2004; Turner et al., 2004), in which learned expectations are violated to produce a prediction error (Corlett et al., 2004). We examined Kamin blocking, in which prior learning interferes with what is subsequently acquired (Kamin, 1969, see Figure 1 for task design). Subjects were asked to imagine themselves working as an allergist confronted with a new patient “*Mr. X”*. Trials composed of presentation of a food picture (representing a meal eaten by *Mr. X*), a predictive button push response by the subject and, following this, an allergic-reaction or no reaction outcome. Subjects held the button down longer the more confident they felt in their prediction (Corlett et al., 2004, 2006; Corlett, Murray et al., 2007), providing a sensitive assay of learning as follows:

Predictivestrength=R×(lengthofbuttonpush)

*R* is the predictive response (coded by +1 for prediction of an allergy and −1 for prediction of no allergy). The blocked cue induces a near zero score, since subjects should not learn about it.

#### 2.5.1 Blocking contingencies in the food-allergy paradigm

To set up blocking, subjects initially learned that a food predicted allergy and then experienced the same food paired with a novel food, this pairing causing an allergy of equivalent magnitude. Under such circumstances, as described above, they should learn little or nothing about the novel food. Finally, during the key phase, blocked novel foods were presented either with an allergic outcome (which would be relatively surprising) or without it (which would be relatively predicted). Comparing the prediction error condition with a well-matched control condition (see Figure 1) enables a quantification of individuals' prediction error signals in key brain regions. This signal has previously served as a prediction error assay enabling us to confirm an associative, PE-driven explanation for the acquisition of causal beliefs (Corlett et al., 2004). In the current study, we were primarily interested in the degree to which it would correlate with schizotypal beliefs and experiences.

#### 2.5.2 Trial sequence

Training consisted of three phases: Learning, in which prior expectancies were developed; Blocking, in which those prior expectancies ‘blocked' new learning; and Violation, which provided a metric for the strength of blocking (see Figure 1 for more information). Subjects saw 10 repetitions of each trial-type during the initial learning phase, they then saw 6 repetitions of each trial-type during the blocking phase and finally six repetitions of each trial-type in the final violation stage. There were filler cues that balanced expectancies about the presence or absence of a predictive relationship across cues (see Table 1 for further clarification). Furthermore, subjects were presented with two independent blocking contingencies across the training stages – one was confirmed at Stage 3 (i.e. the blocked cue did not cause the allergy) and one was violated (the blocked cue was shown causing the allergy). We used the same food stimuli as employed in our prior work (Corlett et al., 2004, 2006; Corlett, Murray et al., 2007; Turner et al., 2004). As before, the roles assigned to particular food cues were counterbalanced across subjects (Corlett et al., 2004, 2006; Corlett, Murray et al., 2007; Turner et al., 2004), as were the relative positions of the foods on screen, such that attentional biases to particular parts of the screen did not develop (Dommett et al., 2005; Kruschke, Kappenman, & Hetrick, 2005).

On each trial, food cues were presented on the screen for 3 s, outcomes for 1 s (see Figure 1). There was a 500 ms inter-trial interval and fixation trials (4 s resting events in which no behavioral response was required and a fixation cross appeared at the center of the screen). These events were presented on average once every ten trials across the task, as in our previous work with this task (Corlett et al., 2004, 2006; Corlett, Murray et al., 2007; Turner et al., 2004).

### 2.6 Behavioral data analysis

We focused our behavioral analyses on confirming that blocking did indeed occur in our subjects. To this end, we planned a paired *t*-test on subjects' prediction confidence for the first trial of stage 3 on which they see the blocked cue alone (mushrooms in Figure 1) compared with their initial prediction the first time they saw the blocking control cue alone (Chili in Figure 1). Mean predictive confidence ratings were calculated such that subjects' responses to the initial presentations of blocking and control cues for both contingencies (confirmed and violated) both contributed to the behavioral analysis. This was legitimate because until this point (i.e. before subjects saw the outcome at the first trial of stage 3), the novelty, familiarity and contingency with the outcome of these parallel causal contingencies were identical.

#### 2.6.1 fMRI data acquisition

We used a Siemens Trio scanner operating at 3 T. A total of 720 gradient echo T2⁎-weighted echo-planar images depicting blood oxygenation level-dependent contrast were acquired for each subject. The first seven images were treated as “dummy” scans and discarded to avoid T1 equilibration effects. The remaining images covered the three task phases that ran continuously, in series: stage 1 (learning) followed by stage 2 (blocking) followed by stage 3 (violation). This was crucial; subjects did not know that there were different learning phases, which encouraged the application of prior learning to current prediction that is so critical to the blocking effect. Images were positioned parallel to the anterior commissure–posterior commissural line and comprised 35 slices, each of 2 mm with a 0.5 mm interslice gap. A repetition time of 1620 ms was used with an echo time of 30 ms and 90° flip angle. The scanner has a 192 mm field of view with a 64×64 data matrix.

#### 2.6.2 fMRI data analysis

fMRI data were analyzed using SPM5 (Wellcome Department of Cognitive Neurology, London, UK; http://www.fil.ion.ucl.ac.uk/spm). The average haemodynamic response to each event was designated at the presentation of the outcome. Trials were modeled using a canonical, synthetic haemodynamic response function (Friston et al., 1998), used as a covariate in a general linear model. A parameter estimate was generated for each voxel for each event. Responses were parametrically modulated by the subjects' confidence in their prediction for that event. Individuals' contrast images, derived from the pair-wise comparisons between key events, were then entered into a second-level group analysis for each of the stages. Given our a priori hypotheses and prior work (Corlett et al., 2006; Corlett, Murray et al., 2007), we used the PickAtlas tool (Maldjian, Laurienti, Kraft, & Burdette, 2003) to confine analyses to a single mask comprised of a series of regions of interest (ROI), total volume 1805 voxels. The five ROIs combined into the mask were: right lateral prefrontal cortex (rPFC, a sphere of radius 10 mm centered on 50, 30, 28 – based on our prior work (Corlett et al., 2004; 2006; Corlett, Murray et al., 2007; Fletcher et al., 2001; Turner et al., 2004), left and right striatum and left and right substantia nigra (defined anatomically using the PickAtlas tool, Maldjian et al., 2003).

Brain responses to events that violated blocking (i.e. events when the blocked cue was shown causing the allergy, *Mushrooms* in Figure 1) were compared with unsurprising control cues (*Chili* in Figure 1). Subjects who blocked most should be most surprised by the blocked cue causing the allergy, indexed as more extensive fronto-striatal activation in response to such trials.

We also identified brain responses to blocking trials (*banana* and *mushrooms,* Figure 1) relative to matched control events (*avocado* and *chillies*, Figure 1). This comparison revealed the brain regions engaged during the blocking process.

### 2.7 Relating PE brain signal to odd beliefs

We aimed to determine the relevance of variability in PE-responsiveness to individual differences in schizotypy, specifically magical ideation and the degree of distress caused by the beliefs captured on the PDI (see above). Therefore, we constructed two separate multiple linear regression models for the Chapman scales and PDI, incorporating regressors for each of the subscales within each statistical model. This allowed us to explore the relationship between PE brain responses and important dimensions of odd beliefs, accounting for the fact that there were subscales that were not pertinent to the present analysis but should nevertheless be included in the model (e.g. social and physical anhedonia from the Chapman scales).

We aimed to determine the relevance of individual PE-responsiveness to schizotypy. Therefore, we computed correlations between phase 3 violation-related activation in the ROI mask and magical ideation and PDI distress (as two separate statistical models), reasoning that weaker blocking would be associated with an attenuated surprise response when the blocked cue was observed causing the allergy (relative to a matched control contingency). We applied small volume correction for multiple comparisons (Worsley et al., 1996). For each correlation we report the *z*-score in the particular regions. All reported findings were associated with false discovery rate corrected *p*-values less than 0.05 (Genovese, Lazar, & Nichols, 2002). For illustrative purposes we plot the relationships between brain responses and behavioral ratings. We are aware of the potential for statistical non-independence or circularity in correlative analysis (Vul & Pashler, 2012) and hence we do not re-compute Pearson's r-values for the relationship between the parameter estimates from our fMRI models and the cognitive measures of interest.

## 3 Results

### 3.1 Peters Delusions Inventory (PDI)

Subjects PDI scores were comparable to published scores for healthy control subjects (Corlett, Simons et al., 2009; Murray, Corlett, & Fletcher, 2010). Their mean number of endorsements on the PDI was five out of 21 questions (*s.d.*=3.1; patients with schizophrenia endorse 10.9 +/− 9.3, Corlett, Simons et al., 2009; Murray et al., 2010). Our subjects' mean distress rating regarding their beliefs was 11.8 (*s.d.*=10.7), their mean pre-occupation with the unusual beliefs they endorsed was 11.9 (*s.d.*=9.6) and their mean conviction regarding those beliefs was 15.5 (*s.d.*=11.4). Again, these values are consistent with prior reports of PDI scores in healthy control subjects (Corlett, Simons et al., 2009; Murray et al., 2010).

### 3.2 Chapman scales

Subjects' scores on the Chapman scales were comparable to prior published work in healthy volunteer subjects (Corlett, Krystal, Taylor, & Fletcher, 2009). Their mean self-reported level of magical ideation was 4.6 (*s.d.*=3.5), mean self-rated perceptual aberration was 3.8 (*s.d.*=4.6), mean physical anhedonia was 8.8 (*s.d.*=4.2) and mean social anhedonia was 5.6 (*s.d.*=4.0).

### 3.3 Blocking behavior

Subjects evidenced behavioral blocking. They were less likely to predict an allergy when confronted with a blocked cue, and their predictions were less confident (*t*=7.169, 2-tailed, *d.f.*=16, *p*<0.0001).

### 3.4 Neural responses to blocking (stage 2)

Blocking trials were associated with an attenuated response in rPFC relative to control trials (*x*=42, *y*=18, *z*=20, *z*-score=2.85, *p*<0.05).

### 3.5 Violation of blocking (stage 3)

Presenting the blocked cue causing the allergy engendered a PE response in rPFC (*x*=42, *y*=18, *z*=20. *z*-score=2.50. *p*<0.05) and bilateral head of caudate (*x*=−6, *y*=16, *z*=6. *z*-score=2.99, *p*<0.05; *x*=4, *y*=14, *z*=6. *z*-score=2.23 *p*<0.05), when compared with control trials.

### 3.6 Relating metrics across stage, brain and behavior

In order to assess the consistency between learned predictions and brain responses across stages 2 and three, first we regressed subjects' behavioral predictions about the blocked cues on trial 1 of stage 3 onto their brain responses during blocking (stage 2). Subjects with the strongest rPFC response to blocking (stage 2) learned inappropriately, predicting allergy following the blocked cue at stage 3 (peak voxel, *x*=42 *y*=36 *z*=16; *z*-score=3.96 *p*<0.05). Second, we regressed stage 2 brain responses during blocking onto stage 3 brain responses to its violation. There was an inverse relationship between PE brain responses to blocking trials at stage 2 and those in response to the violation of blocking at stage 3 (peak voxel, *x*=46 *y*=32 *z*=26; *z*-score=2.60 *p*<0.05). That is, those subjects who activated DLPFC during blocking showed an attenuated surprise response when they observed the blocked cue causing the allergy.

### 3.7 Psychosis-like experiences and prediction error

#### 3.7.1 Magical ideation (Chapman scales)

**Figure 2:**
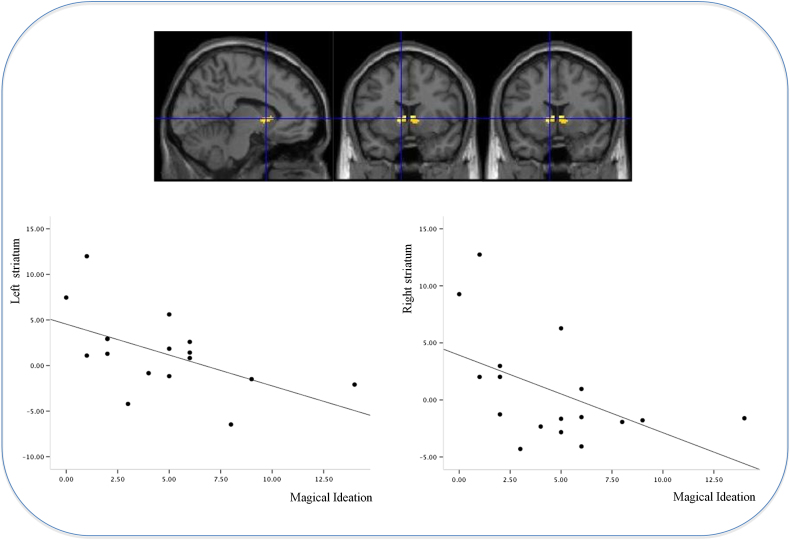
The relationship between striatal prediction error response and magical ideation. Rendering of the relationship between magical ideation score and striatal prediction error signal. Plot on the left depicts the signal (beta-weight parameter estimates) in left striatum (peak voxel: *x*=−16 *y*=16 *z*=2), regressed upon magical ideation score; the right hand plot depicts the beta weights from the peak voxel in the right striatum: *x*=4 *y*=8 *z*=4, regressed upon the magical ideation score.

The severity of subjects' baseline magical ideation correlated negatively with the magnitude of their striatal PE response to the blocked cue causing the allergy; suggesting that subjects reporting most pronounced magical ideation were most likely to have learned inappropriately about the blocked cue (Left: *x*=−16 *y*=16 *z*=2, *z*-score=2.86, *p*<0.05; Right: *x*=4 *y*=8 *z*=4, *z*-score=2.45, *p*<0.05, see Figure 2).

#### 3.7.2 Distress associated with odd beliefs (PDI)

**Figure 3:**
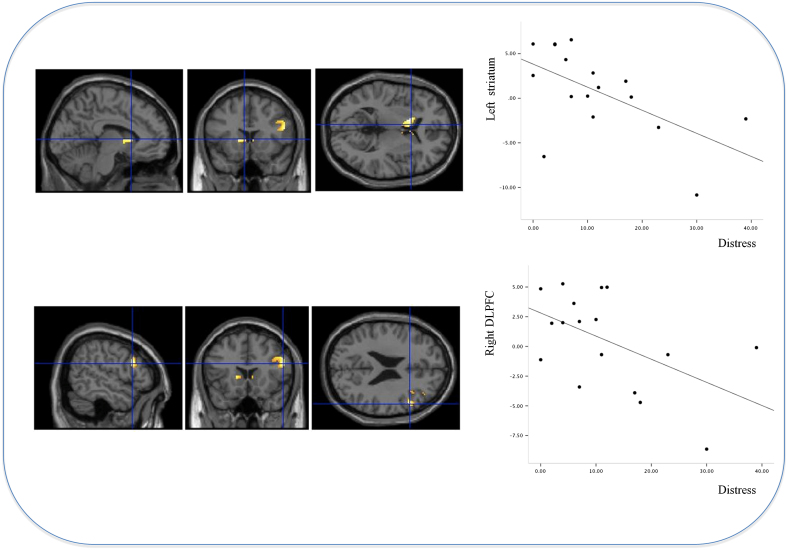
Relating fronto-striatal prediction error signal to distress associated with odd beliefs. Rendering of the relationship between PDI distress score and midbrain, frontal and striatal prediction error signal. Plot depicts the signal in right DLPFC (peak voxel: *x*=54 *y*=18 *z*=24, beta-weights) regressed upon PDI distress score.

PE in the frontal cortex, striatum and midbrain was negatively predictive of the degree of distress that those beliefs caused. That is, people with highest degrees of such distress showed least PE response to violation of blocking-induced expectation in frontal cortex (*x*=54 *y*=18 *z*=24; *z*-score=3.07 *p*<0.05), striatum (*x*=−14 *y*=14 *z*=6; *z*-score=3.01, *p*<0.05) and midbrain (*x*=−14 *y*=−22 *z*=−6; *z*-score=2.97, *p*<0.05, see Figure 3). These results suggest that the subjects who learned inappropriately about the blocked cue (and hence were less surprised when that cue caused the allergy) were more likely to be distressed by their odd beliefs.

### 3.8 Post-hoc analysis – relating PDI distress with stage 2 blocking responses

As a measure of the consistency across learning phases, we explored the relationship between PE response during blocking (which ought to have been attenuated, based on prior learning) and PDI distress score. As predicted, there was a positive relationship between aberrant PE during blocking and PDI distress score. Subjects with inappropriate DLPFC responses during blocking trials were most distressed by their odd beliefs (*x*=44 *y*=34 *z*=38; *z*-score=2.40 *p*<0.05).

### 3.9 Post-hoc analysis – relating other subscales with stage 3 violation responses

For completeness, we explored the relationships between other subscales of the Chapman and Peters scales. No regions correlated with Chapman perceptual aberration, social or physical anhedonia subscales.

Neither endorsement nor pre-occupation showed any relationship with PE brain responses, however, the degree of conviction did correlate with DLPFC prediction error responses. Like distress, there was a negative relationship between belief conviction and DLPFC response at stage 3 (*x*=52, *y*=20, *z*=32, *z*-score=3.16, *p*<0.05) suggesting that those subjects who were less surprised at the blocked cue causing the allergy were more convinced by the odd beliefs that they endorsed.

Given our a priori focus on magical ideation and distress as well as our concern about limiting the number of statistical comparisons, we do not discuss these observations further.

## 4 Discussion

In healthy participants, self-reported unusual beliefs, as well as the distress accompanying them correlated with PE brain signals in ways that overlap intriguingly with our previous observations in the setting of delusional beliefs. Specifically, healthy people reporting such unusual beliefs showed a relative attenuation of brain responses to events that, on the basis of prior experience, ought to be relatively surprising. Given prior observations in people with clinical psychosis (Corlett, Murray et al., 2007; Murray et al., 2008) and in healthy participants under ketamine administration (Corlett et al., 2006), this observation is compatible with the suggestion that unusual, but non-clinical, beliefs show more than just superficial overlap with full psychotic symptoms. The results appear consistent with a continuum model of attenuated psychotic symptoms whereby these unusual mental phenomena represent a milder form of the clinical delusions that attend serious mental illnesses like schizophrenia (Claridge, 1985). In support of this notion, the degree of distress associated with these unusual beliefs was associated with variation in right frontal prediction error signal. What seems to distinguish people who harbor unusual ideas from those who present clinically with delusions is that the latter suffer significantly more distress (Peters et al., 1999b). In the present study, healthy people who held their odd beliefs more like patients (with associated distress) also had prediction error brain responses redolent of patients with clinical delusions (Corlett, Murray et al., 2007).

On the other hand, we also observed a significant association between striatal prediction error signal and the degree of self-reported magical ideation. We have not observed such an association in our work with clinical samples (Corlett, Murray et al., 2007). This result suggests we ought to temper our endorsement of the continuum model slightly. Overall, the findings would be consistent with the following speculation: the striatal PE finding suggests that perhaps healthy unusual ideas have their source in aberrant striatal functioning. Unlike clinical delusions, this level of ideation does not impact upon an individual too detrimentally. Indeed, they may find it personally, socially and even financially advantageous (Nettle & Clegg, 2006). However, if unusual beliefs are associated with inappropriate right frontal cortical dysfunction, more like that of a deluded patient, then belief is associated with a degree of distress. This finding is more consistent with a quasi-dimensional perspective on the relationship between schizotypy and psychosis (Claridge & Beech, 1995). That is, the schizotypal personality measures and striatal prediction error signaling could represent *formes frustes* of clinical psychosis that must interact with other factors such as stress or the consumption of psychotogenic drugs in order to manifest as the full clinical symptom.

Another important factor that distinguishes so-called healthy odd beliefs from delusions is the degree of social support and confirmation that the believer experiences (Peters et al., 1999b). It will be crucial to explore social anhedonia and reduced social interactions as mediating factors in rendering striatally mediated unusual experiences and ideas stressful and therefore pathological in terms of prefrontal function.

These findings are consistent with recent discussions of multiple learning systems in the brain; a striatal controller which represents simple contingencies between events in the world (visual stimuli and the presence of rewards for example) and a more complex prefrontal system which is computationally intense and processes the complicated relationships between those simple contingencies; perhaps even representing a world-model (Daw, Niv, & Dayan, 2005). On the basis of the present data, we argue that a striatal system capable of entertaining irrelevant associations is not necessarily detrimental and, given the link between creativity and healthy schizotypy (Nettle & Clegg, 2006) that we may have a neural signature of individuals' abilities to generate novel or unusual associations (“outside the box” thinking) (de Manzano, Cervenka, Karabanov, Farde, & Ullen, 2010). However, if those novel associations impact upon the prefrontal representation of the world, then the world can become an unpredictable and distressing place, akin to that of a patient with delusional beliefs (Corlett, Krystal et al., 2009; Corlett, Taylor, Wang, Fletcher, & Krystal, 2010).

This explanation shares some surface similarities with Coltheart and colleagues' neuropsychological model of delusions in which delusions are explained with two factors; a deficit in belief evaluation associated with right frontal cortex dysfunction and a deficit in some other system that conveys the delusion's content (Coltheart, 2010; Coltheart, Langdon, & McKay, 2007). Coltheart argues that for the salient experiences that attend delusions in schizophrenia, the striatum represents the neural locus of factor 1 (Coltheart, Langdon, & McKay, 2010). However, we believe that a hierarchical processing model, invoking no clear distinction between experiences and beliefs, provides a more compelling model for understanding fronto-striatal interactions and co-contributions to internal models of the world (Corlett, Taylor et al., 2010). Both regions code prediction error signals (striatum, within a model and prefrontal across models, Waldmann & Martignon, 1998) and it is prediction error function and dysfunction that contributes to healthy and abnormal belief formation (Fletcher & Frith, 2009). This idea of a hierarchically organized system draws on the predictive coding model of neural function in which a primary purpose of neural interactions is to minimize prediction error in pursuit of maximizing the accuracy of predictions of the environment and thus optimizing interactions with it (Friston, 2005). Since prediction error is the driving force in shaping such a system, we argue that schizotypal beliefs can be directly generated, influenced and modified by its alteration.

Given incentive learning theories of psychosis (Kapur, 2003), it is important to consider whether the responses in striatum during our task reflect a reward prediction error response and thus, whether reward prediction errors pertain to delusions (Kapur, 2003) and non-clinical odd beliefs. This is particularly pertinent since it is those striatal responses that relate to healthy magical ideation, which in new religious movement populations has been shown to correlate with rewarding feelings of social inclusion (Peters et al., 1999b). We do not posit a role for reward prediction error in the present results for three reasons: First, the task does not deliver primary or secondary rewards. Second, our study subjects present a striatal signal when they make a prediction and have it violated – they are not garnering reinforcement from making correct responses. Third, theories of striatal function tend to support a role in signaling salience and expectancy violation rather than simply reward. Striatum also responds to punishment (Menon et al., 2007; Romaniuk et al., 2010; Schiller, Levy, Niv, LeDoux, & Phelps, 2008), as well as events that are neither rewarding nor punishing but that are novel or alerting (Zink, Pagnoni, Martin, Dhamala, & Berns, 2003). We do not feel our data examine reward prediction error per se, but rather prediction errors in the context of causal inference (Dickinson, 2001; Redgrave & Gurney, 2006). The present data suggest that the fronto-striatal circuit engaged by this causal inference process (Corlett et al., 2004), is composed of regions differentially related to aspects of healthy odd beliefs in a manner consistent with component regions of the circuit coding different aspects of prediction error driven causal inference (Waldmann & Martignon, 1998). Further, these data provide some insight into the complex relationship between clinical delusions and schizotypal odd beliefs. The specific roles of striatal and frontal prediction error signals in generating and maintaining healthy and problematic beliefs will be an important subject for future studies.

In conclusion, on one hand, our data favor the continuum idea by providing evidence of overlap between prediction error dysfunction in psychosis and in schizotypal beliefs. However, close inspection of our neural data suggests that this is not a simple continuum and that non-clinical odd beliefs may be mediated by a striatal system that is distinct from that which causes pathological delusions (in right frontal cortex). Consistent with this idea, those healthy subjects whose unusual beliefs appeared more like those of patients with psychosis (i.e. being held with greater conviction and accompanied by distress) were more likely to show a patient-like rPFC PE dysfunction (Corlett, Murray et al., 2007). On the other hand, simple endorsement of odd beliefs was not associated with right PFC PE but with striatal prediction error signal, a pattern that has not been observed in patients with endogenous psychosis in the context of causal inference and belief formation.

We acknowledge of course that this is a small sample of subjects. But these admittedly preliminary data demonstrate the potential value of a cognitive neuroscientific approach in exploring links between schizotypy and psychosis and in examining the environmental, neural and cognitive factors that contribute to schizotypy and interact with it to increase the risk of transition to psychotic illness, for example childhood trauma (Dominguez, Saka, Lieb, Wittchen, & van Os, 2010). We note with interest that in an animal model of such developmental trauma, associative blocking (and therefore prediction error processing) was also impaired (Beauchamp, Gluck, Fouty, & Lewis, 1991). Future work could use this relationship between neural prediction error signal and psychopathology to explore the transition from risk to psychosis. Data such as these are likely to have implications for our understanding of psychotic symptoms, our notion of risk for psychosis and perhaps the persistence of psychotic phenotypes despite the fitness costs associated with this illness (David, 2010).

## Figures and Tables

**Table 1:** Summary of task design.

**Stage 1 (learning, 10 repetitions)**	**Stage 2 (blocking, 6 repetitions)**	**Stage 3 (violation, 6 repetitions)**	**Role**
A_1_+	A_1_B_1_+	B_1_+	Violation of Blocking
A_2_+	A_2_B_2_+	B_2_−	Confirmation of blocking
C_1_−	C_1_D_1_+	D_1_+	Control for blocking violation
C_2_−	C_2_D_2_+	D_2_−	Violation of control cue
	EF−	EF−	Stage 2, 2 foods no allergy
	GH−	GH−	Stage 2, 2 foods no allergy
I+	I+	I+	Consistent Allergy
J−	J−	J−	Consistent No Allergy

Letters represent the food cues, a “+” symbol denotes the presence of an allergy and a “−” symbol connotes the absence of allergy following those particular food cues. The contrasts of interest were defined as follows: Blocking (stage 2): ([C_1_D_1_,C_2_D_2_]−[A_1_B_1_,A_2_B_2_]) Blocking violation (stage 3): [B_1_+]−[D_1_+] For the regression analyses: for Stage 2, for each subject a contrast image of blocking trials compared with their control trial ([C_1_D_1_,C_2_D_2_] − [A_1_B_1_,A_2_B_2_]) was entered into the regression model. For Stage 3, we entered contrast images that captured stage 3 blocking violation {[B_1_+]−[D_1_+]} into the regression model.

## References

[bib1] Beauchamp A.J., Gluck J.P., Fouty H.E., Lewis M.H. (1991). Associative processes in differentially reared rhesus monkeys (Macaca mulatta): blocking. Developmental Psychobiology.

[bib2] Chapman J. (1966). The early symptoms of schizophrenia. The British Journal of Psychiatry.

[bib3] Chapman L.J., Chapman J.P., Raulin M.L. (1976). Scales for physical and social anhedonia. Journal of Abnormal Psychology.

[bib4] Chapman L.J., Chapman J.P., Raulin M.L. (1978). Body-image aberration in Schizophrenia. Journal of Abnormal Psychology.

[bib5] Claridge G. (1985). Origins of mental illness.

[bib6] Claridge G., Beech T., Raine A., Lencz T., Mednick S. (1995). Fully and quasi-dimensional constructions of schizotypy. Schizotypal personality.

[bib7] Coltheart M. (2010). The neuropsychology of delusions. Annals of the New York Academy of Sciences.

[bib8] Coltheart M., Langdon R., McKay R. (2007). Schizophrenia and monothematic delusions. Schizophrenia Bulletin.

[bib9] Coltheart M., Menzies P., Sutton J. (2010). Abductive inference and delusional belief. Cognitive neuropsychiatry.

[bib10] Corlett P.R., Aitken M.R., Dickinson A., Shanks D.R., Honey G.D., Honey R.A. (2004). Prediction error during retrospective revaluation of causal associations in humans: fMRI evidence in favor of an associative model of learning. Neuron.

[bib11] Corlett P.R., Taylor J.R., Wang X.J., Fletcher P.C., Krystal J.H. (2010). Toward a neurobiology of delusions. Progress in neurobiology.

[bib12] Corlett P.R., Honey G.D., Aitken M.R., Dickinson A., Shanks D.R., Absalom A.R. (2006). Frontal responses during learning predict vulnerability to the psychotogenic effects of ketamine: linking cognition, brain activity, and psychosis. Archives of General Psychiatry.

[bib13] Corlett P.R., Honey G.D., Fletcher P.C. (2007). From prediction error to psychosis: ketamine as a pharmacological model of delusions. Journal of Psychopharmacology.

[bib14] Corlett P.R., Krystal J.H., Taylor J.R., Fletcher P.C. (2009). Why do delusions persist?. Frontiers in Human Neuroscience.

[bib15] Corlett P.R., Murray G.K., Honey G.D., Aitken M.R., Shanks D.R., Robbins T.W. (2007). Disrupted prediction-error signal in psychosis: evidence for an associative account of delusions. Brain.

[bib16] Corlett P.R., Simons J.S., Pigott J.S., Gardner J.M., Murray G.K., Krystal J.H. (2009). Illusions and delusions: relating experimentally-induced false memories to anomalous experiences and ideas. Frontiers in Behavioral Neuroscience.

[bib17] Corlett, P. R., Taylor, J. R., Wang, X. J., Fletcher, P. C., & Krystal, J. H. (2010). Toward a neurobiology of delusions. *Progress in Neurobiology*. 10.1016/j.pneurobio.2010.06.007 PMC3676875 20558235

[bib18] David A.S. (2010). Why we need more debate on whether psychotic symptoms lie on a continuum with normality. Psychological Medicine.

[bib19] Daw N.D., Niv Y., Dayan P. (2005). Uncertainty-based competition between prefrontal and dorsolateral striatal systems for behavioral control. Nature Neuroscience.

[bib20] de Manzano O., Cervenka S., Karabanov A., Farde L., Ullen F. (2010). Thinking outside a less intact box: Thalamic dopamine D2 receptor densities are negatively related to psychometric creativity in healthy individuals. PloS One.

[bib21] Dickinson A. (2001). The 28th Bartlett Memorial Lecture. Causal learning: an associative analysis. The Quarterly Journal of Experimental Psychology B.

[bib22] Dominguez M.D., Saka M.C., Lieb R., Wittchen H.U., van Os J. (2010). Early expression of negative/disorganized symptoms predicting psychotic experiences and subsequent clinical psychosis: a 10-year study. The American Journal of Psychiatry.

[bib23] Domino E.F., Mirzoyan D., Tsukada H. (2004). N-methyl-D-aspartate antagonists as drug models of schizophrenia: a surprising link to tobacco smoking. Progress in Neuro-Psychopharmacology and Biological Psychiatry.

[bib24] Dommett E., Coizet V., Blaha C.D., Martindale J., Lefebvre V., Walton N. (2005). How visual stimuli activate dopaminergic neurons at short latency. Science.

[bib25] Eckblad M., Chapman L.J. (1983). Magical ideation as an indicator of schizotypy. Journal of Consulting and Clinical Psychology.

[bib26] Fletcher P.C., Anderson J.M., Shanks D.R., Honey R., Carpenter T.A., Donovan T. (2001). Responses of human frontal cortex to surprising events are predicted by formal associative learning theory. Nature Neuroscience.

[bib27] Fletcher P.C., Frith C.D. (2009). Perceiving is believing: a Bayesian approach to explaining the positive symptoms of schizophrenia. Nature reviews Neuroscience.

[bib28] Friston K. (2005). A theory of cortical responses. Philosophical Transactions of the Royal Society of London Series B, Biological Sciences.

[bib29] Friston K.J., Fletcher P., Josephs O., Holmes A., Rugg M.D., Turner R. (1998). Event-related fMRI: characterizing differential responses. Neuroimage.

[bib30] Genovese C.R., Lazar N.A., Nichols T. (2002). Thresholding of statistical maps in functional neuroimaging using the false discovery rate. Neuroimage.

[bib31] Gross G., Huber G. (1972). [Sensory disorders in schizophrenia]. Archiv fur Psychiatrie und Nervenkrankheiten.

[bib32] Johns L.C., van Os J. (2001). The continuity of psychotic experiences in the general population. Clinical Psychology Review.

[bib33] Kamin L., Campbell B.A., Church R.M. (1969). Predictability, surprise, attention, and conditioning. Punishment and aversive Behavior.

[bib34] Kao Y.C., Wang T.S., Lu C.W., Cheng T.H., Liu Y.P. (2012). The psychometric properties of the Peters et al. Delusions Inventory (PDI) in Taiwan: reliability, validity, and utility. Social Psychiatry and Psychiatric Epidemiology.

[bib35] Kapur S. (2003). Psychosis as a state of aberrant salience: a framework linking biology, phenomenology, and pharmacology in schizophrenia. The American Journal of Psychiatry.

[bib36] Kretschmer E. (1925). Physique and character.

[bib37] Kruschke J.K., Kappenman E.S., Hetrick W.P. (2005). Eye gaze and individual differences consistent with learned attention in associative blocking and highlighting. Journal of Experimental Psychology Learning, Memory, and Cognition.

[bib38] Krystal J.H., Petrakis I.L., Limoncelli D., Webb E., Gueorgueva R., D'Souza D.C. (2003). Altered NMDA glutamate receptor antagonist response in recovering ethanol-dependent patients. Neuropsychopharmacology.

[bib39] Laurent A., Biloa-Tang M., Bougerol T., Duly D., Anchisi A.M., Bosson J.L. (2000). Executive/attentional performance and measures of schizotypy in patients with schizophrenia and in their nonpsychotic first-degree relatives. Schizophrenia Research.

[bib40] Linscott R.J., van Os J. (2010). Systematic reviews of categorical versus continuum models in psychosis: evidence for discontinuous subpopulations underlying a psychometric continuum. Implications for DSM-V, DSM-VI, and DSM-VII. Annual Review of Clinical Psychology.

[bib41] Maldjian J.A., Laurienti P.J., Kraft R.A., Burdette J.H. (2003). An automated method for neuroanatomic and cytoarchitectonic atlas-based interrogation of fMRI data sets. Neuroimage.

[bib42] Meehl P.E. (1989). Schizotaxia revisited. Archives of General Psychiatry.

[bib43] Menon M., Jensen J., Vitcu I., Graff-Guerrero A., Crawley A., Smith M.A. (2007). Temporal difference modeling of the blood-oxygen level dependent response during aversive conditioning in humans: effects of dopaminergic modulation. Biological Psychiatry.

[bib44] Moore J.W., Dickinson A., Fletcher P.C. (2011). Sense of agency, associative learning, and schizotypy. Consciousness and cognition.

[bib45] Moore J.W., Dickinson A., Fletcher P.C. (2011). Sense of agency, associative learning, and schizotypy. Consciousness and Cognition.

[bib46] Moran P.M., Al-Uzri M.M., Watson J., Reveley M.A. (2003). Reduced Kamin blocking in non paranoid schizophrenia: associations with schizotypy. Journal of Psychiatric Research.

[bib47] Murray G.K., Corlett P.R., Clark L., Pessiglione M., Blackwell A.D., Honey G. (2008). Substantia nigra/ventral tegmental reward prediction error disruption in psychosis. Molecular Psychiatry.

[bib48] Murray G.K., Corlett P.R., Fletcher P.C. (2010). The neural underpinnings of associative learning in health and psychosis: how can performance be preserved when brain responses are abnormal?. Schizophrenia Bulletin.

[bib49] Nettle D., Clegg H. (2006). Schizotypy, creativity and mating success in humans. Proceedings of the Royal Society: Biological sciences.

[bib50] Peters E., Day S., McKenna J., Orbach G. (1999). Delusional ideation in religious and psychotic populations. British Journal of Clinical Psychology.

[bib51] Peters E., Day S., McKenna J., Orbach G. (1999). Delusional ideation in religious and psychotic populations. The British Journal of Clinical Psychology.

[bib52] Peters E., Joseph S., Day S., Garety P. (2004). Measuring delusional ideation: the 21-item Peters et al. Delusions Inventory (PDI). Schizophrenia Bulletin.

[bib53] Peters E., Joseph S., Day S., Garety P. (2004). Measuring delusional ideation: The 21-item Peters et al. delusions inventory (PDI). Schizophrenia Bulletin.

[bib54] Peters E.R., Joseph S.A., Garety P.A. (1999). Measurement of delusional ideation in the normal population: introducing the PDI (Peters et al. Delusions Inventory). Schizophrenia Bulletin.

[bib55] Petrakis I.L., Limoncelli D., Gueorguieva R., Jatlow P., Boutros N.N., Trevisan L. (2004). Altered NMDA glutamate receptor antagonist response in individuals with a family vulnerability to alcoholism. The American Journal of Psychiatry.

[bib56] Redgrave P., Gurney K. (2006). The short-latency dopamine signal: a role in discovering novel actions?. Nature Reviews Neuroscience.

[bib57] Romaniuk L., Honey G.D., King J.R., Whalley H.C., McIntosh A.M., Levita L. (2010). Midbrain activation during Pavlovian conditioning and delusional symptoms in schizophrenia. Archives of General Psychiatry.

[bib58] Schiller D., Levy I., Niv Y., LeDoux J.E., Phelps E.A. (2008). From fear to safety and back: Reversal of fear in the human brain. The Journal of Neuroscience.

[bib59] Sisti D., Rocchi M.B., Siddi S., Mura T., Manca S., Preti A. (2012). Preoccupation and distress are relevant dimensions in delusional beliefs. Comprehensive Psychiatry.

[bib60] Teufel C., Kingdon A., Ingram J.N., Wolpert D.M., Fletcher P.C. (2010). Deficits in sensory prediction are related to delusional ideation in healthy individuals. Neuropsychologia.

[bib61] Turner D.C., Aitken M.R., Shanks D.R., Sahakian B.J., Robbins T.W., Schwarzbauer C. (2004). The role of the lateral frontal cortex in causal associative learning: exploring preventative and super-learning. Cerebral Cortex.

[bib62] van Os J., Hanssen M., Bijl R.V., Ravelli A. (2000). Strauss (1969) revisited: A psychosis continuum in the general population?. Schizophrenia Research.

[bib63] Vul E., Pashler H. (2012). Voodoo and circularity errors. Neuroimage.

[bib64] Waldmann M.R., Martignon L., Gernsbacher M.A., Derry S.J. (1998). A Bayesian network model of causal learning. Proceedings of the twentieth annual conference of the cognitive science society.

[bib65] Wing J.K., Cooper J.E., Sartorius N. (1974). Measurement and classification of psychiatric symptoms.

[bib66] Worsley K.J., Marrett S., Neelin P., Vandal A.C., Friston K.J., Evans A.C. (1996). A unified statistical approach for determining significant signals in images of cerebral activation. Human Brain Mapping.

[bib67] Zink C.F., Pagnoni G., Martin M.E., Dhamala M., Berns G.S. (2003). Human striatal response to salient nonrewarding stimuli. The Journal of Neuroscience.

